# A combined effort of 11 laboratories in the WHO African region to improve quality of Buruli ulcer PCR diagnosis: The “BU-LABNET”

**DOI:** 10.1371/journal.pntd.0010908

**Published:** 2022-11-04

**Authors:** Estelle Marion, Numfor Hycenth, Sundeep Chaitanya Vedithi, Marie Robbe-Saule, Valérie Donkeng, Line-Marlène Ganlonon, Affolabi Dissou, Solange Kakou Ngazoa, Marie-Jose Kabedi, Arsène Mabika Mabika, Richard Phillips, Michael Frimpong, Dorothy Yeboah-Manu, Vera Yatta Walker, Olaoluwa Akinwale, Maman Issaka, Gisela Bretzel, Kingsley Asiedu, Sara Eyangoh

**Affiliations:** 1 Univ Angers, Nantes Université, INSERM, Immunology and New Concepts in ImmunoTherapy, INCIT, UMR 1302, Angers, France; 2 BU-LABNET coordination center, Centre Pasteur du Cameroun, Yaoundé, Cameroun; 3 Department of Biochemistry, University of Cambridge, Cambridge, United Kingdom & American Leprosy Missions, Greenville, South Caroline, United States of America; 4 Mycobacteriology service, Centre Pasteur du Cameroun, Yaoundé, Cameroun; 5 Centre de Diagnostic et de Traitement de la lèpre et de l’Ulcère de Buruli (CDTLUB)/Raoul et Madeleine Follereau, Pobè, Benin; 6 Laboratoire de Référence des Mycobactéries, Cotonou, Benin; 7 Institut Pasteur, Abidjan, Cote d’Ivoire; 8 Institut National de Recherche Biomédicale, Kinshasa, Rép D. Congo; 9 Unité de Bactériologie, CIRMF, Franceville, Gabon; 10 Kumasi Centre for Collaborative Research in Tropical Medicine, Kwame Nkrumah University of Science and Technology, Kumasi, Ghana; 11 Noguchi Memorial Institute for Medical Research, University of Ghana, Accra, Ghana; 12 National Public Health Reference Laboratory, Liberia Institute of Biomedical Research Charlesville, Liberia; 13 Nigerian Institute of Medical Research, Lagos State, Nigeria; 14 Institut Nationale d’Hygiene, Laboratoire de Biologie Moléculaire, Lomé, Togo; 15 Division of Infectious Diseases and Tropical Medicine, Ludwig-Maximilians University Munich, Germany; 16 Department of Control of Neglected Tropical Diseases, World Health Organization, Geneva, Switzerland; Johns Hopkins University, UNITED STATES

## Abstract

Buruli ulcer is one of the 20 neglected tropical diseases in the world. This necrotizing hypodermitis is a chronic debilitating disease caused by an environmental *Mycobacterium ulcerans*. At least 33 countries with tropical, subtropical and temperate climates have reported Buruli ulcer in African countries, South America and Western Pacific regions. Majority of cases are spread across West and Central Africa. The mode of transmission is unclear, hindering the implementation of adequate prevention for the population. Currently, early diagnosis and treatment are crucial to minimizing morbidity, costs and preventing long-term disability. Biological confirmation of clinical diagnosis of Buruli ulcer is essential before starting chemotherapy. Indeed, differential diagnosis are numerous and Buruli ulcer has varying clinical presentations. Up to now, the gold standard biological confirmation is the quantitative PCR, targeting the insertion sequence IS*2404* of *M*. *ulcerans* performed on cutaneous samples. Due to the low PCR confirmation rate in endemic African countries (under 30% in 2018) for numerous identified reasons within this article, 11 laboratories decided to combine their efforts to create the network “BU-LABNET” in 2019. The first step of the network was to harmonize the procedures and ship specific reagents to each laboratory. With this system in place, implementation of these procedures for testing and follow-up was easy and the laboratories were able to carry out their first quality control with a very high success rate. It is now time to integrate other neglected tropical diseases to this platform, such as yaws or leprosy.

## Author summary

Buruli ulcer is one of the ten skin neglected tropical diseases, caused by *Mycobacterium ulcerans*, and has been reported in over 33 countries worldwide, with Africa bearing about 98% of the disease burden. Prior to antibiotic treatment, the World Health Organization has set a target of at least 70% polymerase chain reaction confirmation rate as target for countries to achieve, yet instead of attaining this target, the confirmation rate has been on a decline in most of these African countries. Procedure ambiguity and low external quality assessment participation were identified as some of the reasons for this drawback. It is against this background that the WHO brought together the efforts of 11 laboratories to form a network termed the “BU-LABNET” in 2019. This manuscript describes the steps taken to harmonize these ambiguous procedures and the provision of reagents to the network laboratories. It also presents the implementation of the new EQA scheme, which is coordinated by one of the African country’s laboratories. The results obtained so far by implementing this strategy is positive and we look forward to extending to other skin NTDs.

## Introduction

Buruli ulcer (BU) is a necrotizing skin disease caused by *Mycobacterium ulcerans (M*. *ulcerans)*. It has been reported in over 33 countries worldwide, with most cases being detected in Western and Central Africa sub-regions. However, only nearly half of these countries regularly report data to the World Health Organization (WHO, 2022). Current options to control BU are limited, as no effective vaccine and preventive methods are available. The knowledge on the mechanism of transmission of the causative agent, *Mycobacterium ulcerans*, is not well known. With the introduction of combination antibiotic therapy, replacing excision surgery as the standard treatment for BU, pre-treatment confirmation of the clinical diagnosis has further gained importance to avoid the redundant use of anti-mycobacterial drugs [1,2]. Of the established tests for the detection of *M*. *ulcerans*, IS*2404* PCR has proven to be the most sensitive and specific, if performed according to demanding quality assurance schemes [3,4]. Therefore, IS*2404* qPCR is currently considered as the gold standard for clinical diagnostic confirmation. WHO thus recommends PCR confirmation before starting treatment due to differential diagnosis, which may detect several non-BU chronic ulcers [5]. Biological confirmation is also important for correct country registration, accurate epidemiology and implementation of management strategies for this neglected tropical disease [6,7].

In 2008, the Technical Advisory Group of the WHO Global BU Initiative recommended the establishment of an external quality assessment (EQA) program for the PCR-based detection of *M*. *ulcerans* in clinical samples. This system was implemented by the Institute of Tropical Medicine (ITM) in Belgium [3] under the coordination of WHO. Despite 4 rounds of this EQAP conducted between 2009 and 2018, *M*. *ulcerans* DNA detection in endemic countries revealed a marked diversity in diagnostic quality. WHO sets at least 70% polymerase chain reaction (PCR) confirmation rate as target for countries to achieve. Most BU endemic countries in the African region have not been able to reach this set target, rather, the rate of confirmation has been declining, from 50 to 20% PCR-confirmed cases in African countries. The declining percentage of BU confirmed cases is attributed to: (i) low rate of PCR confirmation in a number of endemic countries; (ii) long delay in obtaining results from the laboratories; (iii) ambiguity in PCR testing protocols; iv) difficulty in reagent supply and cost. Furthermore, participation in the EQA program by African reference laboratories was low. The Institute of Tropical Medicine (ITM) Antwerp in Belgium discontinued the EQA in March 2019 due to lack of funding to sustain the EQA program. At the moment, it was imperative that a novel EQA program be rapidly introduced to improve the performance of laboratories involved in the molecular diagnosis of BU in the endemic countries in Africa, this to ensure that (i) patients receive quality diagnostic results and (ii) data recorded by WHO is accurate, reliable and comparable with those of other continents such as Australia. WHO then envisaged the creation of a network of African laboratories coordinated by one of its members. To realize this project, financial support from NGOs was sought to ensure its success.

In this context, 11 laboratories of 9 endemic countries (Benin, Cameroon, Côte d’Ivoire, Democratic Republic of Congo, Gabon, Ghana, Liberia, Nigeria and Togo), supported by WHO, American Leprosy Missions (ALM), Fondation Raoul Follereau (FRF) in France, as well as external experts, formed a resilient network of Buruli ulcer laboratories (BU-LABNET). Prior to implementation of the EQA program, the objectives of this network were (i) to harmonize and disseminate standard operating procedures (SOPs) to member laboratories, (ii) provide the member laboratories with the same reagents for PCR testing (to harmonize testing protocols) and (iii) provide member laboratories with technical assistance for qPCR implementation and analysis of results. Here, we aim to describe the efforts put in place to improve the quality of BU diagnosis in African countries. This work confirms that reliable IS*2404* PCR results can only be generated through strict adherence to good clinical laboratory practices and implementation of quality assurance protocols.

## Material and methods

**Fig 1:**
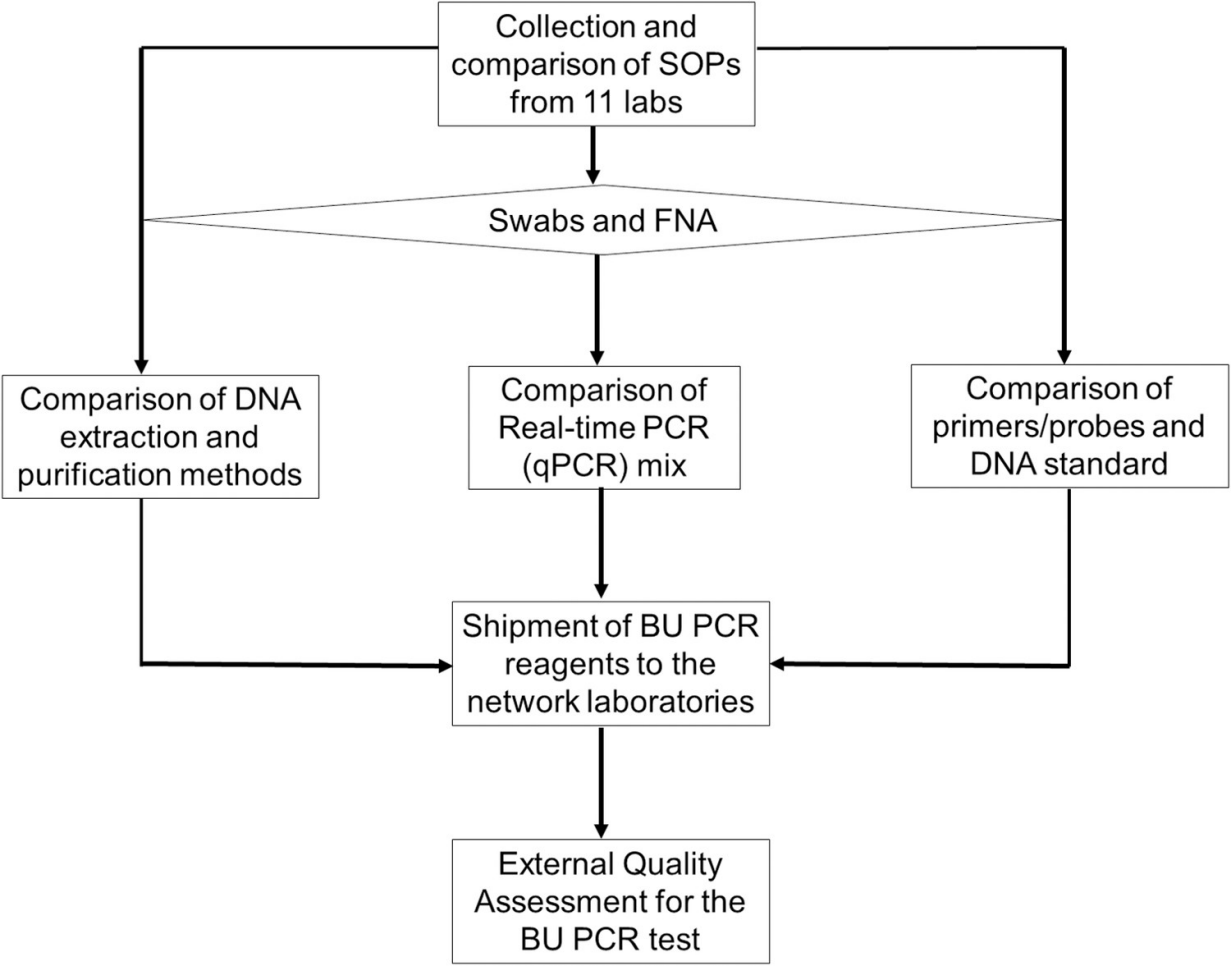
Flowchart of study design.

This study was conducted following the design in the represented in the flowchart in **Fig 1.**

### Collection and comparison of SOPs

The 11 laboratories of the network were chosen based on available WHO information on their capacity to perform qPCR, the number of reported BU cases in their respective countries and their willingness to join the network. Centre Pasteur du Cameroun (CPC) was designated as the network coordinating center by WHO, based on their operational capacity and previous good performance in the Institute of Tropical Medicine (ITM) EQA program. A request was made and all 11 laboratories sent their PCR procedures to CPC including information on qPCR machine, extraction method, type of master mix used, DNA standard, and available equipment, etc. The different protocols were compared based on the following criteria: feasibility (ease to implement), turnaround time, contamination risk during sample processing, cost effectiveness, robustness, sensitivity and specificity. A comparison based on the DNA extraction/purification method, the qPCR procedure including probes/primers, and use of the DNA standard was performed. Samples tested by the laboratories included swabs, Fine Needle Aspirates (FNA) and biopsies. Since biopsies are no longer recommended (too invasive), only swabs and FNA were used to validate the selected methods.

### Comparison of DNA Extraction and Purification methods

From the 11 SOPs received by CPC, we listed 4 main methods of DNA extraction used: 1) Automatized DNA extraction using the Maxwell Instrument (Promega), used by 2 laboratories: 2) Commercial kits; Gentra Puregene kit (as stated in the Buruli ulcer Manual for Health Care Providers) and QIAamp DNA mini kit with an enzymatic lysis step (Qiagen), used by 4 laboratories: 3) Alkaline lysis method using “in-house” buffers [8] and a commercial kit [9] (GenoLyse, Hain LifeScience) used by 4 other laboratories, and 4) The Modified Boom method [10] used by a single laboratory.

The first two methods have been discarded as too expensive, too long and with an enzymatic lysis step not adapted to lyse mycobacterial cell wall. We decided to focus on the alkaline lysis procedure, which is known to be a robust, rapid and inexpensive method based on lysis of bacterial cell walls with sodium hydroxide (NaOH) and heat. This method was or was not associated with a purification step using a QIAquick DNA purification kit (Qiagen) based on a silica membrane transfer to eliminate any inhibitors for qPCR assay. We chose to compare the alkaline lysis procedure from the “in house” SOP and the commercial kit with and without the purification step. Evaluation of the DNA extraction methods was performed using four swabs and four FNA samples with known concentration of *M*. *ulcerans*.

### Comparison of Real-time PCR (qPCR) mix, primers/probes and DNA standard

The 11 laboratories are equipped with different types of thermocyclers and use a large diversity of qPCR mixes. The challenge was to choose a qPCR mix adaptable to all thermal cyclers, and, if possible, validated for room temperature shipment and with a reasonable cost. After a corresponding literature review and contact with different suppliers, both qPCR mixes containing low ROX (adaptable to all thermocyclers) were compared and evaluated for sensitivity and efficacy: the Brillant II QPCR Master Mix (Agilent) and the 5X HOT FIREPol Probe Universal qPCR Mix (Solis BioDyne). In parallel, two primer/probe sets (**Table 1**) were tested, using both amplification reagents and the *M*. *ulcerans* DNA controls. Some laboratories used different *M*. *ulcerans* DNA as standards for quantification and positive controls. Others did not use it at all. The DNA used was purified from *M*. *ulcerans* isolates of infected mouse tissues, or from an IS*2404* plasmid (S1 Data). We compared the DNA standard generated from a mouse tail infected with *M*. *ulcerans* (strain 1G897), mimicking the DNA found in human samples for diagnosis and the DNA plasmid (GenExpress) which was tested as an external homologous DNA standard of known copy number to produce standard curves for IS*2404*. The former was serially diluted (1:10) in sterile water over a concentration range of 6.4x10^6^ to 6.4x10^0^ U/mL while the latter was diluted over a concentration range of 1x10^7^ to 1x10^0^ U/mL for testing. Each 10-fold dilution was performed in duplicates for n = 3 experiments and the standard curve generated by a linear regression of the threshold cycle (Ct) against the logarithm of *M*. *ulcerans* DNA concentration. The qPCR efficiency (E) was calculated from the slope of the standard curve using the classical formula E = 10^(-1/slope)^ -1.

**Table 1:** Sequences of primers and probes used during the SOP harmonization procedure.

Primers/probes	Sequences (5’-3’)	
IS*2404*_F = MuF1	ATTGGTGCCGATCGAGTTG	Set 1 (Rondini et al. 2003) [11]
IS*2404*_R = MuR1	TCGCTTTGGCGCGTAAA	
IS*2404*_P = MuFB	FAM—CACCACGCAGCATTCTTGCCGT—BHQ1	
IS*2404*_TF	AAAGCACCACGCAGCATCT	Set 2 (Fyfe et al. 2007) [12]
IS*2404*_TR	AGCGACCCCAGTGGATTG	
IS*2404*_TP	FAM—CCGTCCAACGCGATCGGCA—BHQ1	

### Shipment of BU PCR reagents to the network laboratories

After the SOPs had been harmonized and validated, the network supplied the selected and tested reagents by a DHL shipment to each laboratory at room temperature. These included extraction kits, qPCR master mix, lyophilized primers and probe, and lyophilized DNA for the standard curve. The laboratories were required to use the provided reagents to test samples for routine diagnosis and for the EQA program.

### External quality assessment for the BU PCR test–Proficiency testing (PT)

Proficiency testing involved the distribution of coded specimens with known status to the network laboratories, with the coding sequence differing from laboratory to laboratory. For the proficiency testing of clinical samples, coded specimens with known content were distributed by CPC to the 11 laboratories that were asked to process the samples using DNA extraction and PCR procedures according to the BU-LABNET SOPs (Table 2). The proficiency testing panel consisted of 10 suspensions of recent clinical known BU negative samples spiked with different concentrations of heat deactivated positive suspensions of fresh BU culture isolates (Table 3). All suspensions were added to PBS and ethanol (50% v/v, 35% final ethanol concentration) to inactivate bacteria. The panel consisted of the following samples: 4 BU PCR negatives, 1 drug-sensitive Mycobacterium tuberculosis (MTB), 1 non-TB Mycobacteria (NTM) and 4 BU PCR positives. The 4 positive suspensions represented a variety of weak and high (mean Ct of 25.87, 30.17, 34.03 and 37.13) positive *M*. *ulcerans* DNA. All samples were aliquoted in tight-capped 1.5ml cryotubes, making a panel of 10 PT materials for each member laboratory. In total, 16 panels were prepared, which included at least three backup sets of the PT panel, and stored at 4°C at the coordinating center. A panel was first tested at the BU-LABNET coordinating center laboratory by 2 qualified laboratory technicians using the standardized extraction and amplification methods with same reagents used by the other BU-LABNET member laboratories. This was done immediately after the samples were prepared. To test for stability, a panel was tested, stored at 4°C, and same samples tested again one month after. Meanwhile, for shipment quality, a panel was subjected to different temperatures for different periods and then tested. After preparation, panel samples were stored at 4°C for 24 hours and tested afterwards. The same samples were transferred to a 37°C incubator for 24 hours and then retested. Shipment was done by DHL at room temperature according to international IATA regulations (non-infectious laboratory material). Participating laboratories were asked to process the EQA panel using the BU-LABNET reagents and developed SOPs. Each panel was accompanied by testing instructions and a result sheet. Results were returned as positive or negative, with the cycle threshold value reported for the positive results.

**Table 2:** Participants in the first round BU-LABNET EQA program.

CPC-2021-EQAP-Mulc _01	
Date of panel distribution	28/05/2021
Number of participants	11
Number of countries	9
Number of respondents	10
Number of respondents that submitted quantitative data	10

**Table 3:** Qualitative results in the first round BU-LABNET EQA program.

Laboratory code	# concordant results/Total # suspensions tested (%)	# false positives (%)	# false negatives (%) among strong positives	# false negatives (%) among weak positives	#positive results obtained (%) for the MTB and NTM samples	Number of clerical errors	TAT- time from sample reception to submision of results *(days)*
3	10/10 (100%)	0/4 (0%)	0/2 (0%)	0/2 (0%)	0/2 (0%)	0	14 days
4	7/10 (70%)	3/4 (75%)	0/2 (0%)	0/2 (0%)	1/2 (50%)	0	20 days
5	10/10 (100%)	0/4 (0%)	0/2 (0%)	0/2 (0%)	0/2 (0%)	NI	24 days
6	10/10 (100%)	0/4 (0%)	0/2 (0%)	0/2 (0%)	0/2 (0%)	NI	35 days
7	10/10 (100%)	0/4(0%)	0/2 (0%)	0/2 (0%)	0/2 (0%)	0	23 days
8	7/10 (70%)	0/4(0%)	1/2 (50%)	2/2 (100%)	0/2 (0%)	NI	28 days
9	10/10 (100%)	0/4(0%)	0/2 (0%)	0/2 (0%)	0/2 (0%)	NI	24 days
10	9/10 (90%)	0/4(0%)	0/2 (0%)	1/2 (50%)	0/2 (0%)	NI	51 days
11	10/10 (100%)	0/4(0%)	0/2 (0%)	0/2 (0%)	0/2 (0%)	NI	19 days
12	10/10 (100%)	0/4(0%)	0/2 (0%)	0/2 (0%)	0/2 (0%)	0	17 days

NI: No Information

## Results

### Network launch and selection of procedures

In October 2019, 11 laboratories from 9 African countries (**Table 4**) met at the CPC, Cameroon, and adopted the BU-LABNET charter (www.africabulabnet.org). From the review and comparison of all the SOPs, only 1 out of the 11 laboratories used gel-based PCR, while the others performed testing on the real time PCR (qPCR) procedure. To avoid contamination and to achieve more sensitivity, only the qPCR procedure is recognized as the gold standard method for BU confirmation. The laboratory using the gel-based PCR assay had to switch to an available qPCR machine in the institute in order to be part of the BU-LABNET. Some equipment/material and infrastructural requirements were needed to belong to the network and for implementation of the procedures: 3 distinct PCR rooms-including a wash room, precision pipettes, a refrigerator and freezer in each room, a microbiological safety cabinet and a dry bath.

**Table 4:** Laboratories from 9 African countries and activities in 2020 and 2021.

				2020	2020	2021	2021
Country	City	Agency/Institution	Lab status	number of requested PCR analyses	Number of positive samples	number of requested PCR analyses	Number of positive samples
Benin	Pobè	Centre de Diagnostic et de Traitement de l’Ulcère de Buruli	Public	267	79	245	77
Cameroon	Yaounde	Centre Pasteur of Cameroon, Yaoundé	Parapublic	56	24	399	51
Cote d’Ivoire	Abidjan	Institut Pasteur de Côte d’Ivoire	Parapublic	500	239	444	239
Democratic Rupublic of Congo	Kinshasa	Institut National de Recherche Biomédicale	Public	173	31	254	67
Gabon	Franceville	Unité de Bactériologie, CIRMF	Public	13	4	29	7
Ghana	Kumasi	Kumasi Centre for Collaborative Research	Public, Research	216	37	201	32
Ghana	Accra	Noguchi Memorial Institute for Medical Research	Research	199	20	143	11
Liberia	Monrovia	National Public Health Reference Laboratory	Public	-	-	125	1
Nigeria	Lagos	Nigerian Institute of Medical Research	Research	19	0	94	11
Togo	Lomè	National Institute of Hygiene	Public	68	34	34	14
		**Total**		1511	468(40%)	1968	510(26%)

### Sampling and storage

Samples tested by the 11 laboratories included swabs, FNA and biopsies. However, biopsies were excluded from the procedure. The use of storage medium for transportation was also prohibited as culture is not recommended to confirm BU and not useful for the clinician decision. Details are available in the SOP1 for sampling and storage (***SOP1***: ***S****ampling*, *storage and transport*) (S2 Data). Also detailed in SOP2 is the procedure for storage before analysis (***SOP2*:** *Treatment of the samples in the laboratory*: *registration*, *procedure of storage before analysis*) (S3 Data).

### Selection of extraction method

**Fig 2:**
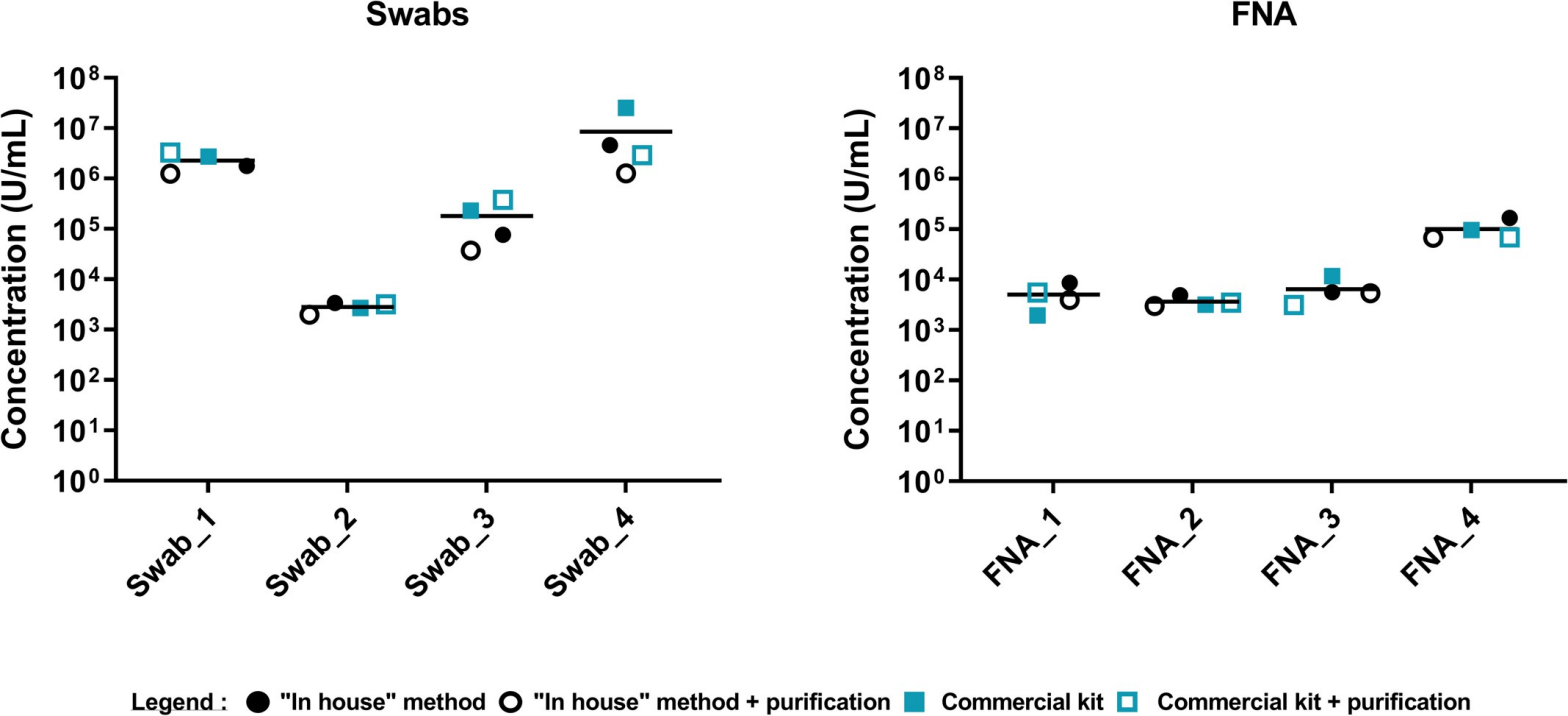
Comparison results of DNA extraction methods. **(A)** from four swabs, and **(B)** from four FNA. No significant difference was observed between the “in-house” method and the commercial kit (Dunn’s multiple comparisons test).

**Fig 3:**
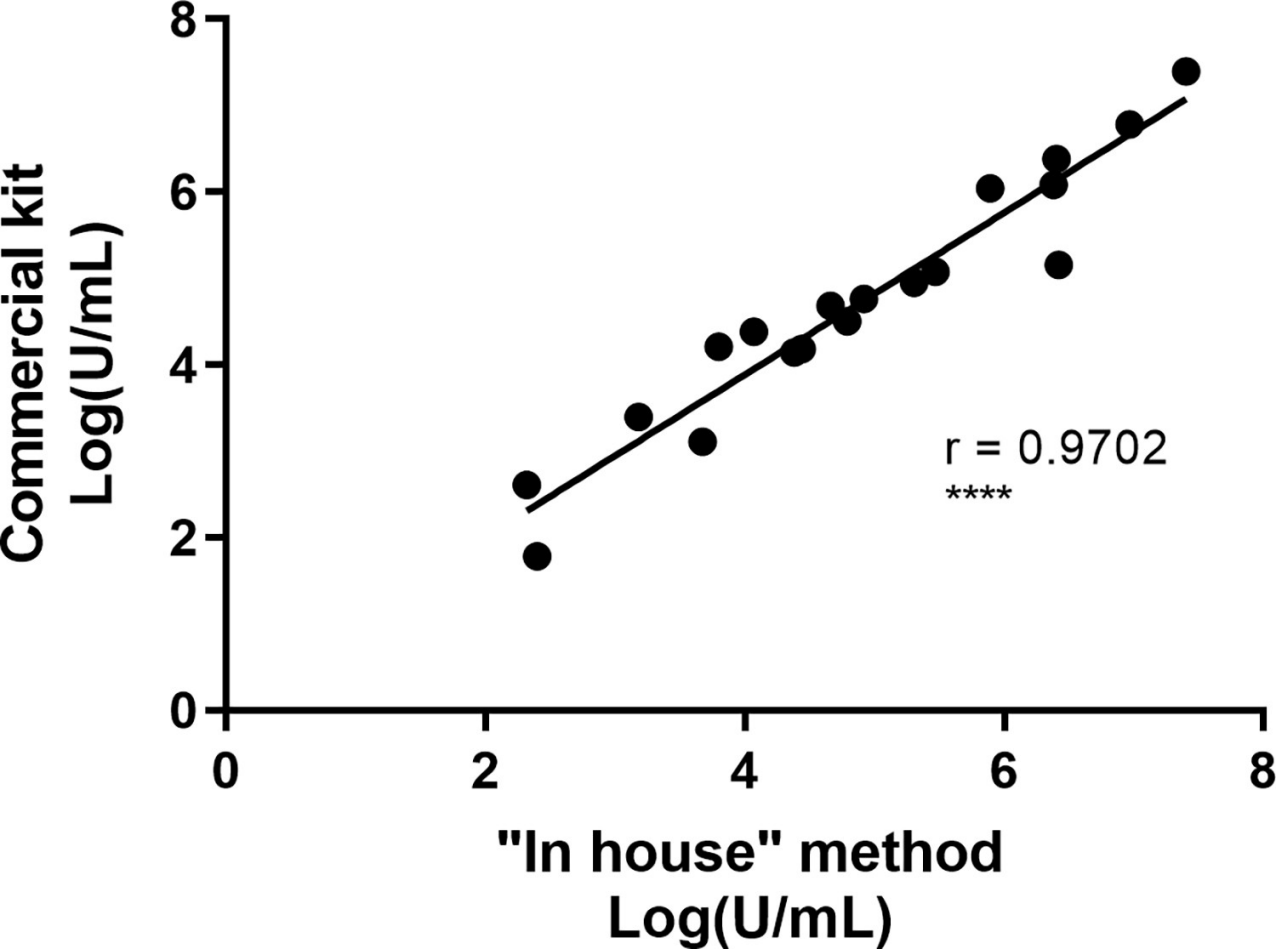
Validation of the DNA extraction method using commercial kit (GenoLyse). Nineteen positive samples were extracted with the “in house” method followed by purification on Qiagen column versus the commercial kit. Validation of the commercial kit for DNA extraction was evaluated with Spearman’s correlation test. Each dot corresponds to a sample. The x and y axes indicate the logarithm of *M*. *ulcerans* DNA concentration. Spearman’s correlation coefficient = 0.9702. ****, Correlation was statistically significant with p<0.0001. No significant difference was observed between the “in-house” method and the commercial kit (Wilcoxon test).

Comparison of the “in house” and commercial kit for alkaline lysis with or without the purification step on silica membrane shows that there is no significant difference in term of quantity of qPCR detected bacteria by any of the four methods (**Fig 2**). We then tested 19 additional swabs and FNA samples using the GenoLyse kit and we obtained similar results to the ‘in house’ method (**Fig 3**). Spearman’s coefficient of correlation between these methods (r = 0.9702) indicated similar results and a statistically significant correlation of p<0.0001. It allowed us to select the commercial kit without purification as the standard procedure for DNA extraction of *M*. *ulcerans* from swabs and FNA samples. It will facilitate shipment, and the standardization amongst all the laboratories. The procedure for DNA extraction from human samples is detailed in SOP3 (***SOP3*:** *Extraction and purification of DNA for M*. *ulcerans detection*) (S4 Data)

### Selection of the qPCR master mix reagents

**Fig 4:**
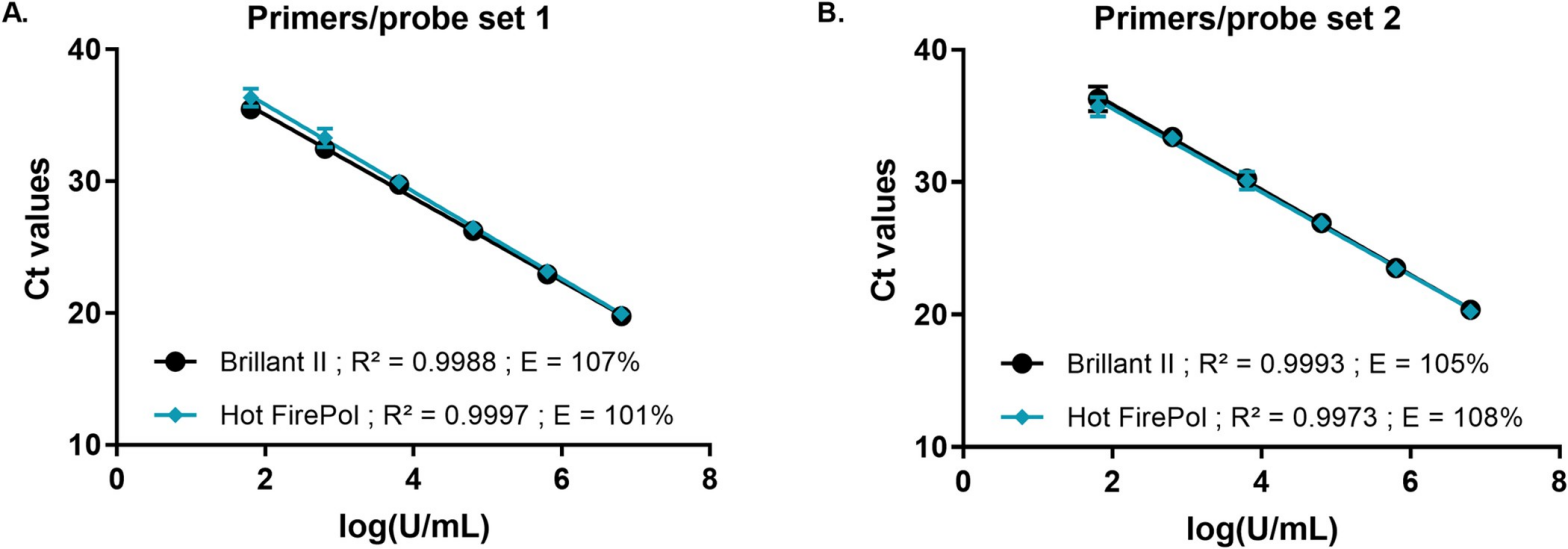
Comparison of qPCR mixes and primers/probe sets for sensitivity and efficiency. Serial 10-fold dilution from 6.4x10^6^U/mL to 6.4x10^0^U/mL of *M*. *ulcerans* DNA. The standard curve was generated by a linear regression of the threshold cycle (Ct) against the logarithm of *M*. *ulcerans* DNA concentration. Each 10-fold dilution was performed in duplicate for n = 3 experiments, one standard deviation (SD) is shown. **(A)** Primers/probe set 1: Brillant II (*y* = −3.16*x* + 41.37); Hot FirePol (*y* = −3.307*x* + 42.44). **(B)** Primers/probe set 2: Brillant II (*y* = −3.217*x* + 42.31); Hot FirePol (*y* = −3.147*x* + 41.84).

**Fig 5:**
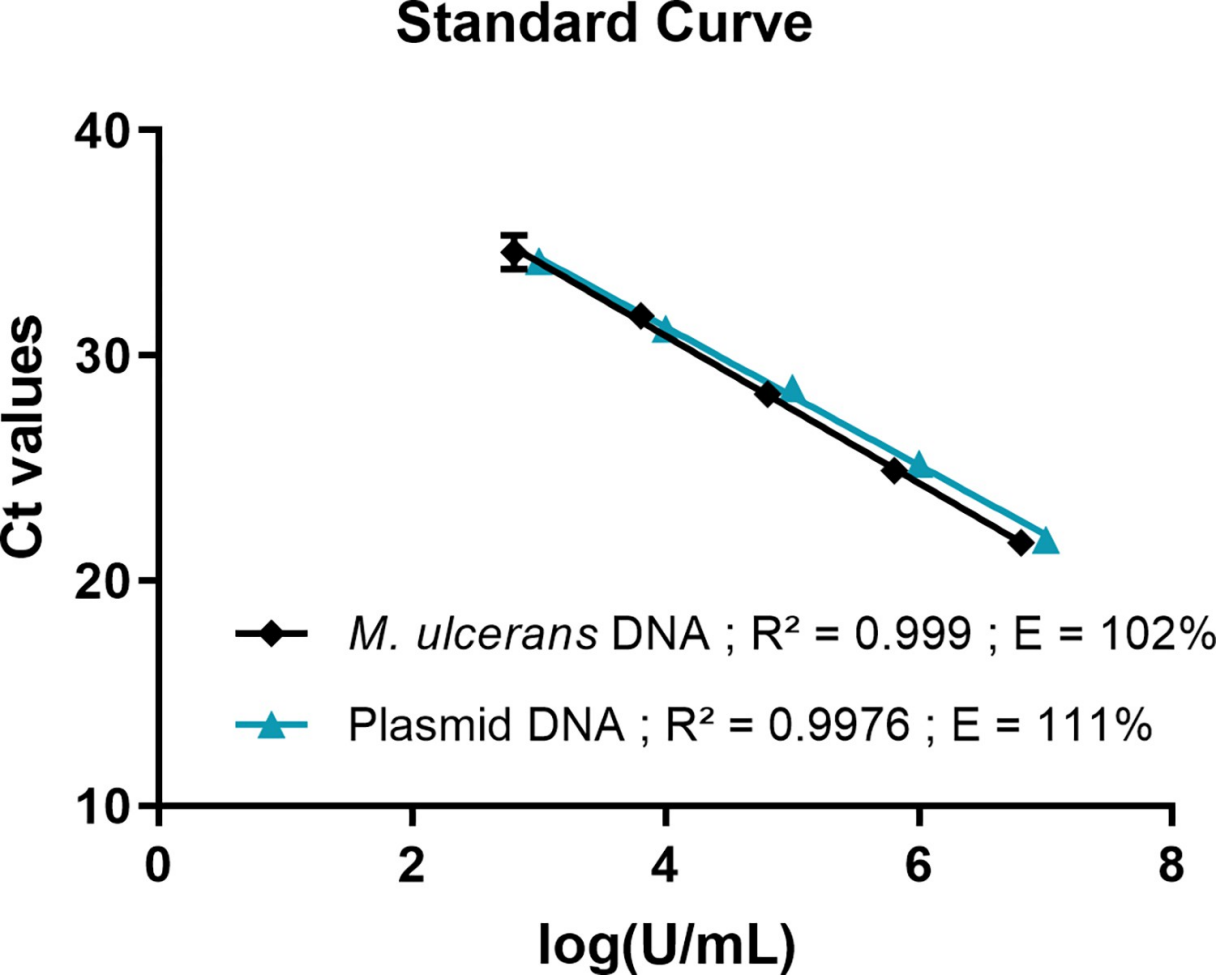
Comparison of internal positive control: Standard curve for IS*2404*. Serial 10-fold dilution from 6,4x10^6^U/mL to 6.4x10^0^U/mL of *M*. *ulcerans* DNA (in black). Serial 10-fold dilution from 1x10^7^U/mL to 1x10^0^U/mL of plasmid DNA (in blue). The standard curve was generated by a linear regression of the threshold cycle (Ct) against the logarithm of DNA concentration. Each 10-fold dilution was performed in duplicate, one standard deviation (SD) is shown. *M*. *ulcerans* DNA standard curve (*y* = −3.271*x* + 43.96); Plasmid standard curve (*y* = −3.074*x* + 43.56).

Furthermore, we compared the efficiency of two different master mixes: Brillant II qPCR Master Mix and HOT FIREPol Probe Universal qPCR Mix. An efficiency of 107% was obtained for the Brillant II qPCR Master Mix and 101% for the HOT FIREPol Probe Universal qPCR Mix using primers/probe set 1 (**Fig 4A**). Similar results were observed with primers/probe set 2 (**Fig 4B**); an efficiency of 105% using the Brillant II qPCR Master Mix and 108% with the HOT FIREPol Probe Universal qPCR Mix. The two master mix are comparable; however, we decided to select the HOT FIREPol Probe Universal qPCR Mix for BU-LABNET laboratories because it is guaranteed stable at room temperature for up to a month, ideally suitable for a shipment at room temperature from Europe to African countries. Furthermore, this master mix is cheaper compared to other master mixes. It was observed that the efficiency of both primers used by the laboratories in the BU-LABNET is comparable. However, the primers/probe set 2 was adopted because of the absence of a difference in the single nucleotide polymorphisms (SNPs) with the reference genome. Finally, standard curves obtained from *M*. *ulcerans* DNA and the plasmid standard IS*2404* had an almost similar PCR efficiency; 102% and 111% respectively (**Fig 5**). Since the plasmid is commercially standardized with a similar concentration, it was adopted and validated for *M*. *ulcerans* quantification to be used by all laboratories in the network. It will facilitate quantification and comparison amongst the laboratories. The procedure for preparation of the qPCR mix is available in the SOP4 and a work sheet for each PCR run was also developed (***SOP4*:** *Q-PCR protocol*, *master mix preparation*, *analysis of the results + worksheet for each PCR run)* (S5 Data).

### Standard operating procedures

After validation of the protocols by the network advisory board, the standardized procedures were disseminated to all the laboratories. Also available on the BU-LABNET website at https://www.africabuLABNET.org/index.php/en/resources/harmonized-sops, and online on the WHO website: https://www.who.int/publications/i/item/9789240007222. As of now, all laboratories in the network have switched to the new DNA extraction and qPCR methods for BU routine diagnosis.

### External quality assessment results

The 11 laboratories having received the reagents from BU-LABNET, participated in the first EQA program round (**Table 2**), but one of them did not send back results. In addition, an associate member laboratory participated in the program. Before performing the EQA program, the 11 laboratories performed a PCR assay using the supplied plasmid and reagents. The amplification curves were sent to the advisory board to ensure that the amplifications ran correctly with the new reagent in the thermocycler of each lab. Of the 10 participating African laboratories that completed the EQA, 7 had 100% concordant results, 1 with 90% concordant results, and 2 with 70% concordant results (**Table 3**). Participants required an overall score of 90% or greater to demonstrate satisfactory performance. One laboratory had false positive results for 3 samples, while 2 laboratories had false negatives. Among these 2 laboratories, 1 had false negatives among both strong and weak positives, while the other had a false negative among weak positive; all indicating a sensitivity problem. Of the 10 participating laboratories, only 4 provided information that facilitated review for clerical errors, and of these laboratories, no clerical error was detected. Finally, 8/10 laboratories respected the defined deadline of 30 days after PT panel receipt.

## Discussion

This initial work allowed for the successful standardization of laboratory protocols in response to resolving the procedure ambiguity issues experienced by the network laboratories and to ensure sustainability. To note, a WHO BU PCR laboratory manual already exists (edited by Françoise Portaels in 2014). However, almost none of the laboratories adhered to the procedures outlined in this manual, probably due to the cost and complexity. In addition, the previous EQA programs did not provide all the necessary assistance because each laboratory used different SOPs and reagents. The creation of the BU-LABNET will allow for the simplification and harmonization of procedures and an efficient quality control because individual/laboratory level coaching is possible and improvement is facilitated.

Proposed SOPs and methods are simple and efficient, reproducible and adapted to the African context (shipment at room temperature, short extraction time). All countries have accepted the different procedures and were provided with reagents through the BU-LABNET; first shipment in December 2020 and the second in September 2021. The partnership was signed for an initial period of 5 years and a shipment of reagents is scheduled every year, quantity adapted to the laboratory workload 2 years before creation of the network. The BU-LABNET spends about 15000 euros, equivalent to 5000 PCR reactions per year (3 euros/PCR), including reagent shipments (100–120 euros/shipment). Efficiency of implemented activities depends on the responsibility of the country BU National Programs to organize the transfer of samples to the laboratories and to support the operational and structural costs.

This first EQA program was necessary to implement the new protocols after the standardization of BU PCR procedures and methods. From the results of this EQA, the false negatives could probably be attributed to problems originating from the extraction procedure and/or pipetting techniques. Meanwhile, implications of the false positive and false negative results of respondent laboratories may be that patients suffering from diseases other than BU receive inappropriate treatment or patients with BU are wrongly considered as being affected by other diseases. Individual feedback was provided to the respective laboratories in a confidential manner bearing in mind that the EQA program is intended only for improvement purposes.

In line with quality assurance, laboratories that scored <80% in the scheme benefitted from online troubleshooting and mentoring from network experts, including a completely new prepared batch of samples shipped to these laboratories. This new batch consisted of 4 samples; 1 negative sample (water) and 3 BU positive suspensions of *M*. *ulcerans* DNA (with mean Ct values of 28.42, 31.59 and 34.72). All samples were aliquoted in tight-capped 1.5ml cryotubes, making a panel of 4 PT materials, and shipped through DHL to each of the laboratories.

A remarkable performance improvement (100%), was experienced after the laboratories tested the new batch of shipped samples. Also, onsite assessment visits have been planned to all the laboratories as part of the ongoing capacity building objective to enhance the continued high performance of the network laboratories.

Since its commencement in 2019, 2 new laboratories have joined the BU-LABNET: West African Center for Cell Biology and Infectious Pathogens (WACCBIP) in Ghana (an academic laboratory) and St Joseph’s Hospital Adazi Nnukwu, Nigeria (private institution). Additionally, in August 2021, personnel from two laboratories (National Public Health Laboratory in Congo Brazzaville and the Institute Pasteur Bangui, Central African Republic) received a one-week BU PCR training at CPC, and this was conducted entirely using the standardized SOPs. The prerequisite for becoming a member in the network for these capacitated laboratories would be for them to report the number of BU PCR analyses performed every quarter for up to a year. To stay as an active member and continue to receive reagents for qPCR confirmation, laboratories have to continue testing samples regularly. Now that laboratories are ready, national programs and NGOs must invest in communication between hospitals and laboratories and ensure that the delivery of samples becomes frequent and reliable. Laboratories have a maximum of 7 days to deliver results upon receipt of samples.

Despite the COVID-19 pandemic in 2020, we note that some laboratories continued diagnosis BU through PCR confirmation (**Table 4**). In 2021, this activity steadily increased and for 2022 so far, the number of cases diagnosed by the labs have significantly increased. We observe that the PCR positivity rate of suspected cases is less than 50% in all laboratories, supporting the importance of testing for the bacillus before starting treatment.

It is evident, given the standardization of processes and the commitment and engagement of all members and partners of this novel network, that there will be a significant increase in the quality of BU diagnostic results delivered to patients, leading to an increased qPCR confirmation rate. In addition, from participating in the EQA scheme, the laboratories will improve their diagnostic performance a great deal. In the future, the network envisages more visibility and scale up to involve other countries in the African continent. The vision of the BU-LABNET is to integrate other skin NTDs on to the molecular platform in the different member laboratories starting with yaws that is targeted for eradication. The strategy used for BU will be implemented equally for yaws.

## Supporting information

S1 Data Plasmid IS2404. (PDF)

S2 Data SOP1: Sampling, storage and transport. (PDF)

S3 Data SOP2: Treatment of the samples in the laboratory: registration, procedure of storage before analysis. (PDF)

S4 Data SOP3: Extraction and purification of DNA for M. ulcerans detection. (PDF)

S5 Data SOP4: Q-PCR protocol, master mix preparation, analysis of the results + worksheet (after confirmation of master mix, probe and primers) for each PCR run. (PDF)

## References

[1] Chauty A, Ardant MF, Marsollier L, Pluschke G, Landier J, Adeye A, et al. Oral treatment for Mycobacterium ulcerans infection: results from a pilot study in Benin. Clin Infect Dis. 2011;52(1):94–6. doi: 10.1093/cid/ciq072 . 21148526

[2] Phillips RO, Robert J, Abass KM, Thompson W, Sarfo FS, Wilson T, et al. Rifampicin and clarithromycin (extended release) versus rifampicin and streptomycin for limited Buruli ulcer lesions: a randomised, open-label, non-inferiority phase 3 trial. Lancet. 2020;395(10232):1259–67. doi: 10.1016/S0140-6736(20)30047-7 ; PubMed Central PMCID: PMC7181188. 32171422 PMC7181188

[3] Eddyani M, Lavender C, de Rijk WB, Bomans P, Fyfe J, de Jong B, et al. Multicenter external quality assessment program for PCR detection of Mycobacterium ulcerans in clinical and environmental specimens. PLoS One. 2014;9(2):e89407. doi: 10.1371/journal.pone.0089407 ; PubMed Central PMCID: PMC3931755. 24586755 PMC3931755

[4] Lavender CJ, Fyfe JA. Direct detection of Mycobacterium ulcerans in clinical specimens and environmental samples. Methods Mol Biol. 2013;943:201–16. doi: 10.1007/978-1-60327-353-4_13 . 23104291

[5] Toutous Trellu L, Nkemenang P, Comte E, Ehounou G, Atangana P, Mboua DJ, et al. Differential Diagnosis of Skin Ulcers in a Mycobacterium ulcerans Endemic Area: Data from a Prospective Study in Cameroon. PLoS Negl Trop Dis. 2016;10(4):e0004385. doi: 10.1371/journal.pntd.0004385 ; PubMed Central PMCID: PMC4830608. 27074157 PMC4830608

[6] Simpson H, Deribe K, Tabah EN, Peters A, Maman I, Frimpong M, et al. Mapping the global distribution of Buruli ulcer: a systematic review with evidence consensus. Lancet Glob Health. 2019;7(7):e912–e22. doi: 10.1016/S2214-109X(19)30171-8 ; PubMed Central PMCID: PMC6614043. 31200890 PMC6614043

[7] Simpson H, Tabah EN, Phillips RO, Frimpong M, Maman I, Ampadu E, et al. Mapping suitability for Buruli ulcer at fine spatial scales across Africa: A modelling study. PLoS Negl Trop Dis. 2021;15(3):e0009157. doi: 10.1371/journal.pntd.0009157 ; PubMed Central PMCID: PMC7959670. 33657104 PMC7959670

[8] Marion E, Ganlonon L, Claco E, Blanchard S, Kempf M, Adeye A, et al. Establishment of quantitative PCR (qPCR) and culture laboratory facilities in a field hospital in benin: 1-year results. J Clin Microbiol. 2014;52(12):4398–400. doi: 10.1128/JCM.02131-14 ; PubMed Central PMCID: PMC4313327. 25320228 PMC4313327

[9] Frimpong M, Ahor HS, Sakyi SA, Agbavor B, Akowuah E, Phillips RO. Rapid Extraction Method of Mycobacterium ulcerans DNA from Clinical Samples of Suspected Buruli Ulcer Patients. Diagnostics (Basel). 2019;9(4). doi: 10.3390/diagnostics9040204 ; PubMed Central PMCID: PMC6963521. 31779247 PMC6963521

[10] Affolabi D, Sanoussi N, Vandelannoote K, Odoun M, Faihun F, Sopoh G, et al. Effects of decontamination, DNA extraction, and amplification procedures on the molecular diagnosis of Mycobacterium ulcerans disease (Buruli ulcer). J Clin Microbiol. 2012;50(4):1195–8. doi: 10.1128/JCM.05592-11 ; PubMed Central PMCID: PMC3318535. 22259213 PMC3318535

[11] Rondini S, Mensah-Quainoo E, Troll H, Bodmer T, Pluschke G. Development and application of real-time PCR assay for quantification of Mycobacterium ulcerans DNA. J Clin Microbiol. 2003;41(9):4231–7. doi: 10.1128/JCM.41.9.4231-4237.2003 ; PubMed Central PMCID: PMC193839. 12958250 PMC193839

[12] Fyfe JA, Lavender CJ, Johnson PD, Globan M, Sievers A, Azuolas J, et al. Development and application of two multiplex real-time PCR assays for the detection of Mycobacterium ulcerans in clinical and environmental samples. Appl Environ Microbiol. 2007;73(15):4733–40. doi: 10.1128/AEM.02971-06 ; PubMed Central PMCID: PMC1951036. 17526786 PMC1951036

