# Supplementary material for: A combined effort of 11 laboratories in the WHO African region to improve quality of Buruli ulcer PCR diagnosis: The “BU-LABNET”
Source: PLoS Negl Trop Dis. 2022 Nov 4;16(11):e0010908. doi: 10.1371/journal.pntd.0010908 (PMC9668193; doi:10.1371/journal.pntd.0010908)
Supplement: S1 Data — (PDF) [file pntd.0010908.s001.pdf]

Plasmid Standard

Organism	**Mycobacterium ulcerans (IS2404)**	plasmid No.	**30-8606-01**
		insert length	**643 bp**

cloned fragment:
5´-CCAGTGAGAAGACCTTCCGCGCTGTTTTGTCTCGGCTAGACCCCGCCGACCTCAACG CCAGGATGGGCAGTTACTTCACTGCACACGTGGCCAGCAGCGACCCCAGTGGATTGGT GCCGATCGCGTTGGACGGCAAGATGCTGCGTGGTGCTTTACGCGCCAAAGCGACAGCC ACGCATCTCGTGTCGGTGTTCGCCCACCGTGCCCGATTGGTGCTCGGTCAACTCGCTGT CGCCGAGAAAAGCAATGAAATTCCCTGCGTACGTGCCCTGCTCACGCTGCTACCGGAT AACTTGCGGTGGCTGGTCACCGTGGATGCGATGCATACCCAGGTCGTCACCGCGAAGT TGATCTGCGCCACCTTGAAGTCGCACTACCTGATGATCGTCAAGTCCAACCAAGCCAA AATACTTGCCCGTATCACCGCGCTGCCCTGGGCCGAGGTGCCCGCAGCCGCTACCGAC GACTCCCGCGGCCACGGCCGTGTCGAGACCCGCACCCTGCAAATCATCACCGCTGCAC GAGGAATCGGCTTCCCCTACGCAAAACAAATCATCCGGATCACTCGTGAACGCTTGAT CACCGCCACCGACCAGCGCAGCGTGGAGGTGGTCTATGCCATCTGCAGCCTGCCGTTC GAGCA-3´

Green: primers
Red: probe

Blast result NCBI:

Mycobacterium ulcerans Agy99, complete genome

Sequence ID: CP000325.1 Length: 5631606
Range 1: 2135492 to 2136134

Alignment statistics for match #1

Score	Expect	Identities	Gaps	Strand
1182 bits(640)	0.0	642/643(99%)	0/643(0%)	Plus/Plus

```
Query   1        CCAGTGAGAAGACCTTCCGCGCTGTTTTGTCTCGGCTAGACCCCGCCGACCTCAACGCCA  60
                 ||||||||||||||||||||||||||||||||||||||||||||||||||||||||||||
Sbjct   2135492  CCAGTGAGAAGACCTTCCGCGCTGTTTTGTCTCGGCTAGACCCCGCCGACCTCAACGCCA  2135551

Query   61       GGATGGGCAGTTACTTCACTGCACACGTGGCCAGCAGCGACCCCAGTGGATTGGTGCCGA  120
                 ||||||||||||||||||||||||||||||||||||||||||||||||||||||||||||
Sbjct   2135552  GGATGGGCAGTTACTTCACTGCACACGTGGCCAGCAGCGACCCCAGTGGATTGGTGCCGA  2135611
```

Plasmid Standard

```
Query  121      TCGCGTTGGACGGCAAGATGCTGCGTGGTGCTTTACGCGCCAAAGCGACAGCCACGCATC  180
                ||||||||||||||||||||||||||||||||||||||||||||||||||||||||||||
Sbjct  2135612  TCGCGTTGGACGGCAAGATGCTGCGTGGTGCTTTACGCGCCAAAGCGACAGCCACGCATC  2135671

Query  181      TCGTGTCGGTGTTCGCCCACCGTGCCCGATTGGTGCTCGGTCAACTCGCTGTCGCCGAGA  240
                ||||||||||||||||||||||||||||||||||||||||||||||||||||||||||||
Sbjct  2135672  TCGTGTCGGTGTTCGCCCACCGTGCCCGATTGGTGCTCGGTCAACTCGCTGTCGCCGAGA  2135731

Query  241      AAAGCAATGAAATTCCCTGCGTACGTGCCCTGCTCACGCTGCTACCGGATAACTTGCGGT  300
                ||||||||||||||||||||||||||||||||||||||||||| ||||||||||||||||
Sbjct  2135732  AAAGCAATGAAATTCCCTGCGTACGTGCCCTGCTCACGCTGCTGCCGGATAACTTGCGGT  2135791

Query  301      GGCTGGTCACCGTGGATGCGATGCATACCCAGGTCGTCACCGCGAAGTTGATCTGCGCCA  360
                ||||||||||||||||||||||||||||||||||||||||||||||||||||||||||||
Sbjct  2135792  GGCTGGTCACCGTGGATGCGATGCATACCCAGGTCGTCACCGCGAAGTTGATCTGCGCCA  2135851

Query  361      CCTTGAAGTCGCACTACCTGATGATCGTCAAGTCCAACCAAGCCAAAATACTTGCCCGTA  420
                ||||||||||||||||||||||||||||||||||||||||||||||||||||||||||||
Sbjct  2135852  CCTTGAAGTCGCACTACCTGATGATCGTCAAGTCCAACCAAGCCAAAATACTTGCCCGTA  2135911

Query  421      TCACCGCGCTGCCCTGGGCCGAGGTGCCCGCAGCCGCTACCGACGACTCCCGCGGCCACG  480
                ||||||||||||||||||||||||||||||||||||||||||||||||||||||||||||
Sbjct  2135912  TCACCGCGCTGCCCTGGGCCGAGGTGCCCGCAGCCGCTACCGACGACTCCCGCGGCCACG  2135971

Query  481      GCCGTGTCGAGACCCGCACCCTGCAAATCATCACCGCTGCACGAGGAATCGGCTTCCCCT  540
                ||||||||||||||||||||||||||||||||||||||||||||||||||||||||||||
Sbjct  2135972  GCCGTGTCGAGACCCGCACCCTGCAAATCATCACCGCTGCACGAGGAATCGGCTTCCCCT  2136031

Query  541      ACGCAAAACAAATCATCCGGATCACTCGTGAACGCTTGATCACCGCCACCGACCAGCGCA  600
                ||||||||||||||||||||||||||||||||||||||||||||||||||||||||||||
Sbjct  2136032  ACGCAAAACAAATCATCCGGATCACTCGTGAACGCTTGATCACCGCCACCGACCAGCGCA  2136091

Query  601      GCGTGGAGGTGGTCTATGCCATCTGCAGCCTGCCGTTCGAGCA  643
                |||||||||||||||||||||||||||||||||||||||||||
Sbjct  2136092  GCGTGGAGGTGGTCTATGCCATCTGCAGCCTGCCGTTCGAGCA  2136134
```
