# Supplementary material for: A combined effort of 11 laboratories in the WHO African region to improve quality of Buruli ulcer PCR diagnosis: The “BU-LABNET”
Source: PLoS Negl Trop Dis. 2022 Nov 4;16(11):e0010908. doi: 10.1371/journal.pntd.0010908 (PMC9668193; doi:10.1371/journal.pntd.0010908)
Supplement: S2 Data — (PDF) [file pntd.0010908.s002.pdf]

Standard Operating Protocol 1 :
Recommendation for sampling, transport and storage of samples for Buruli ulcer diagnosis
With a worksheet for request Buruli ulcer confirmation by PCR

Author	Reviewer	Authorizer
Numfor Hycenth<br>Estelle Marion<br>Sara Eyangoh	BU Lab network members	Advisory Board

ABREVIATIONS

BU	Buruli Ulcer
PCR	Polymerase Chain Reaction
WHO	World Health Organization

Table of Contents

I. PURPOSE 2

II. APPLICATION DOMAIN 2

III. ASSOCIATED DOCUMENTS 2

IV. TYPE OF SAMPLES 2

V. REAGENTS AND CONSUMABLES 3

VI. EQUIPMENT 3

VII. PATIENT INFORMATION 3

VIII. STORAGE CONDITIONS 3

IX. INTERNAL QUALITY CONTROL (IQC) 3

X. SAFETY PRECAUTIONS 3

XI. REFERENCE 4

XII. READING AND UNDERSTANDING LIST 4

Annex 1: List of material and reagent 5

Standard Operating Protocol 1 :
Recommendation for sampling, transport and storage of samples for Buruli ulcer diagnosis
With a worksheet for request Buruli ulcer confirmation by PCR

I. PURPOSE

This Standard Operating Protocol (SOP) aims to present the recommendations of sampling, transport and storage of clinical specimens collected for confirmation of Buruli ulcer by Polymerase Chain Reaction (PCR). For diagnostic purposes, samples should be collected before treatment. Given the heterogeneous distribution of mycobacteria in lesions, at least two clinical specimens should be collected from each lesion.

II. APPLICATION DOMAIN

To be applied to all laboratory members of the BU LAB for the PCR diagnosis of Buruli ulcer

III. ASSOCIATED DOCUMENTS

Worksheet for request for Buruli ulcer confirmation by PCR

IV. TYPE OF SAMPLES

► Swabs are used for sampling of opened lesions with undermined edges.

► Fine needle aspiration (FNA) is used for sampling of closed lesions or open wounds with closed edges (not undermined).

► Biopsy is not recommended for case confirmation of Buruli ulcer.

4.1 Swabs

- Use a sterile unitary swab
- Swab under the undermined edge of the ulcer
- After swabbing, replace the swab in the original tube. Do not add any liquid.

  A minimum of 2 swabs are required per lesion. If there are several lesions, realize 2 swabs/lesion.

4.2 Fine needle aspiration

- Transfer 0.5ml of sterile water in the microtube with screw-cap or vacutainer blood dry tube (red cap).

  Alternatively, you can use physiological water or PBS. Do not inoculate the liquid into the lesion.
- Using a 23G needle and a syringe of 2ml, aspirate the liquid from the closed lesion.
- Put the content of the syringe into the microtube or vacutainer tube containing sterile water.
- Gently draw some of the liquid into the needle and then expel it back into the microtube. To ensure that all of the sample is transferred, repeat this three times, then close the microtube.

  NB: Do not inoculate liquid into the lesion.
- Use a new syringe and needle to repeat aspiration in case of large lesions.

V. REAGENTS AND CONSUMABLES

See list in annex1

VI. EQUIPMENT

Not applicable

VII. PATIENT INFORMATION

All samples should be hermetically closed, identified with the date of the sampling, the first and surname of the patient, with a permanent marker.

If a patient has two types of samples (ie a fine needle and swabs for example) or several lesions, consider 2 different samples to be analyzed.

Shipment needs to be accompanied by an information sheet with a table summarizing the samples. See ***worksheet for request for Buruli ulcer confirmation by PCR***

VIII. STORAGE CONDITIONS

8.1 Storage before transportation

Store the samples at 2°C to 8°C or otherwise at room temperature in a dry place.

The maximum waiting time before transportation is within one week/ maximum two weeks if large countries.

8.2 Storage during transportation to the laboratory

Samples are shipped at room temperature or in a cool box if available.

8.3 Storage at the laboratory

Upon arrival at the laboratory, the samples must be stored at 2°C to 8°C until treatment for PCR analysis. See SOP2.

IX. INTERNAL QUALITY CONTROL (IQC)

Not applicable

X. SAFETY PRECAUTIONS

Always consider all used materials as infectious and discard appropriately.
Discard all needles in a safety box/sharps container

XI. REFERENCE

1. Laboratory diagnosis of Buruli ulcer: A WHO Manual for Health-care providers (edited by: Françoise Portaels) 2014. Available at *https://apps.who.int/iris/handle/10665/111738*; accessed on 28-11-19

XII. READING AND UNDERSTANDING LIST

NAME OF PERSONNEL	DATE	SIGNATURE

<u>Annex 1:</u> List of material and reagents for sampling, transport and storage of samples for Buruli ulcer diagnosis

Name	reference	commentary
Swabs: Sterile and in a tube	For example: 8150CC (Gauss); 552C (Copan)	Numerous references exist. Ensure swabs are individually pack in a tube
Needles and syringes	21G to 23 G needles<br>1ml to 2ml syringes	
1.5ml microtube with screw-cap	For example: 39289 (Dutscher)	Ensure that cap is attached to the tube
Vacutainer dry tube (red cap)		If no screw-cap tube available
Sterile water	Not applicable	Not applicable
Permanent marker	Not applicable	Ball-point tip
Worksheet for BU confirmation request	Use worksheet disseminated by BU lab network	Worksheet version?? (*To ensure everyone uses the same version)*
Waste container for discarding swabs	Not applicable	Leak proof
Sharps container/ safety box for discarding needles	Not applicable	Leak proof and puncture-resistant (*the picture is an example of a safety container)*
Transport box (optional)	Not applicable	Tight fitting lid
Refrigerator to store samples (optional)	Not applicable	2-8°C
Gloves	Not applicable	Non powdered
