# Supplementary material for: A combined effort of 11 laboratories in the WHO African region to improve quality of Buruli ulcer PCR diagnosis: The “BU-LABNET”
Source: PLoS Negl Trop Dis. 2022 Nov 4;16(11):e0010908. doi: 10.1371/journal.pntd.0010908 (PMC9668193; doi:10.1371/journal.pntd.0010908)
Supplement: S3 Data — (PDF) [file pntd.0010908.s003.pdf]

Standard Operating Protocol 2:
Registration and Treatment of samples prior to PCR analysis for Buruli ulcer

Author	Reviewer	Authorizer
Numfor Hycenth<br>Estelle Marion<br>Sara Eyangoh	BU Lab network members	Advisory Board

ABREVIATIONS

BU	Buruli Ulcer
PCR	Polymerase Chain Reaction
WHO	World Health Organization

Table of Contents

I. PURPOSE 2

II. APPLICATION DOMAIN 2

III. ASSOCIATED DOCUMENTS 2

IV. TYPE OF SAMPLES 2

V. REAGENTS AND CONSUMABLES 2

VI. EQUIPMENT 2

VII. REGISTRATION 2

VIII. PROCEDURE OF SAMPLE PROCESSING 3

IX. INTERNAL QUALITY CONTROL (IQC) 3

X. SAFETY PRECAUTIONS 3

XI. REFERENCE 3

XII. READING AND UNDERSTANDING LIST 4

Annex 1: List of material and reagent 5

Standard Operating Protocol 2:
Registration and Treatment of samples prior to PCR analysis for Buruli ulcer

I. PURPOSE

This Standard Operating Protocol (SOP) aims to present the recommendations of registration and treatment of samples for Buruli ulcer diagnosis by PCR.

II. APPLICATION DOMAIN

To be applied to all laboratory members of the BU LAB Network for the PCR diagnosis of Buruli ulcer

III. ASSOCIATED DOCUMENTS

BU Manual register
BU request form

IV. TYPE OF SAMPLES

► Swabs are used for sampling of opened lesions with undermined edges

► Fine needle aspiration (FNA) is used for sampling of closed lesions or open wounds with closed edges (not undermined).

► Biopsy is not recommended for case confirmation of Buruli ulcer.

V. REAGENTS AND CONSUMABLES

See list in annex1

VI. EQUIPMENT

See list in annex 1

VII. REGISTRATION

When a shipment for BU confirmation is received in the lab, proceed to registration and treatment of the samples within the day or the day just after.

For registration, complete the manual register (optional: informatics register, but manual register has to be present in any case). Manual register is composed of the following information present in the worksheet BU request:

- Name of the structure requesting the PCR analysis
- Name of the patient
- Surname of the patient
- Sampling date
- Sampling type
- Lab sample number
- PCR data
- PCR result
- Number of Bacilli/ml

Keep the request sheet received with the shipment in a folder.

Attribute a “Lab sample number” to each new sample. Keep continuity between the samples.

VIII. PROCEDURE OF SAMPLE PROCESSING

NB: Each tube must be identified with the "Lab sample number".

8.1. Dry Swabs

- Rehydrate swabs in a 15ml tube containing 2ml of sterile water and vortex.
  Normally, there are at least two swabs per lesion. Pool all swabs of a same lesion in a single 15ml tube.
- Wait at least 5 minutes and vortex again.
- Gently discard the swabs from the 15ml tube
- Pipet and transfer 400µl in a 1.5ml microtube with screw cap. - Transfer the rest in a 1.5ml microtube with screw cap. Use it for Ziehl-Neelsen staining and store the rest as a backup at -20°C.

8.2 Fine needle aspiration (FNA)

- Vortex the tube containing the FNA
- Pipet 400µl and transfer them in a microtube with screw cap for DNA extraction.

If the volume is <500µl, add 500µl of water to the FNA before pipetting. Store the rest as backup at -20°C.

Keep the tubes containing 400µl of DNA suspension at room temperature for DNA extraction (SOP3) or freeze it at -20°C until DNA extraction.

IX. INTERNAL QUALITY CONTROL (IQC)

Not applicable

X. SAFETY PRECAUTIONS

Always consider all used materials as infectious and discard appropriately.
Discard all needles in a safety box/sharps container

XI. REFERENCE

1. Laboratory diagnosis of Buruli ulcer: A WHO Manual for Health-care providers (edited by: Françoise Portaels) 2014. Available at *https://apps.who.int/iris/handle/10665/111738*; accessed on 28-11-19

XII. READING AND UNDERSTANDING LIST

NAME OF PERSONNEL	DATE	SIGNATURE

Annex 1: List of material and reagent for registration and Treatment of samples before PCR analysis

Name	reference	commentary
A manual register	Not applicable	Obtained locally
BU request form	Not applicable	Format specific to country
50ml Falcon tubes (PP, graduated, conical bottom, blue screw cap, sterile)	Not applicable<br>For example 227 261 Greiner bio one)	Withstand centrifugation
15ml Falcon tubes (PP, graduated, conical bottom, blue screw cap, sterile)	Not applicable<br>For example 188261 Greiner bio one	Withstand centrifugation
Sterile water	Not applicable	Not applicable
1.5ml microtube with screw-cap	For example: 39289 (Dutscher)	Ensure that cap is attached to the tube
Storage box	Not applicable	Plastic box preferable
Vortex mixer	Not applicable	Currently used in respective labs
Pipettes	Not applicable	Currently used in respective labs
Filter tips	Will be provided by the BU LABNET, based on pipette information by labs	Labs will maintain currently used pipettes
Gloves	Not applicable	Non powdered
Disposable lab coat	Not applicable	Long sleeves/full length
-20°C Freezer	Not applicable	To store DNA suspension prior to extraction
Waste container	Not applicable	Leak proof
