# Supplementary material for: A combined effort of 11 laboratories in the WHO African region to improve quality of Buruli ulcer PCR diagnosis: The “BU-LABNET”
Source: PLoS Negl Trop Dis. 2022 Nov 4;16(11):e0010908. doi: 10.1371/journal.pntd.0010908 (PMC9668193; doi:10.1371/journal.pntd.0010908)
Supplement: S4 Data — (PDF) [file pntd.0010908.s004.pdf]

Standard Operating Protocol 3:
Extraction and Purification of DNA from *M. ulcerans* (without Internal Positive Control)

Authors	Reviewers	Authorizer
Numfor Hycenth<br>Estelle Marion<br>Sara Eyangoh	BU Lab network members	Advisory Board

ABREVIATIONS

BU	Buruli Ulcer
PCR	Polymerase Chain Reaction
WHO	World Health Organization

Table of Contents

I. PURPOSE 2

II. APPLICATION DOMAIN 2

III. ASSOCIATED DOCUMENTS 2

IV. TYPE OF SAMPLES 2

V. REAGENTS AND CONSUMABLES 2

VI. EQUIPMENT 2

VII. PROCEDURE 2

VIII. INTERNAL QUALITY CONTROL (IQC) 3

IX. SAFETY PRECAUTIONS 3

X. REFERENCE 3

XI. READING AND UNDERSTANDING LIST 4

Annex 1: List of material and reagent 5

Standard Operating Protocol 3:
Extraction and Purification of DNA from *M. ulcerans* (without Internal Positive Control)

I. PURPOSE

This Standard Operating Protocol (SOP) aims to present the different steps allowing DNA extraction and purification of samples for PCR targeting *Mycobacterium ulcerans*.

II. APPLICATION DOMAIN

To be applied to all laboratory members of the BU LAB Network for the PCR diagnosis of Buruli ulcer

III. ASSOCIATED DOCUMENTS

None

IV. TYPE OF SAMPLES

► Swabs are used for sampling of opened undermined lesions.

► Fine needle aspiration (FNA) is used for sampling of closed lesions or opened but not undermined.

► Biopsy is not recommended for case confirmation of Buruli ulcer.

V. REAGENTS AND CONSUMABLES

See list in annex1

VI. EQUIPMENT

See list in annex 1

VII. PROCEDURE

7.1 DNA EXTRACTION

DNA extraction is performed by bacterial lysis through Alkaline lysis using the Genolyse kit (Ref 51610, Hain LifeScience)

1. For each sample, use 400µl of specimen suspension prepared previously and placed in a microtube with screw cap (see in SOP2).
2. Centrifuge the tube at 12000g for 15min at RT
3. Discard delicately the supernatant using a P1000 filter tip. A pellet can be visible or not.
4. Resuspend the pellet with 400µl of water and centrifuge at 12000g for 15min at RT
5. Discard delicately the supernatant
6. Resuspend the pellet in 50µl of A-LYS buffer
7. Incubate 10min at 95°C
8. Centrifuge tube for 10 sec to pellet the suspension
9. Add 50µl of Buffer A-NB to neutralize
10. keep tube at 4°C if use in the day for PCR amplification or store at -20°C

NB: For swabs and FNA samples, no need to perform purification step

7.2 DNA PURIFICATION FOR BIOPSY SAMPLES

DNA purification is recommended in cases of biopsy samples to remove traces of inhibitors.

Use the "QIAquick purification kit" (ref 28106, QIAGEN) as following :

1. Add 300µl of PB buffer to each sample and 10µl of Na Acetate 3M
2. Load the column with the sample and centrifuge 1min, 5000g, RT
3. Wash the column with 750µl of PE buffer and centrifuge 1min 5000g RT
4. Repeat a second centrifugation without adding a buffer in order to remove any trace of ethanol
5. Eluate DNA by adding 100µl of EB buffer and centrifuge 1min 5000g RT
6. Store at 4°C for short time storage or at -20°C for long time storage.

VIII. INTERNAL QUALITY CONTROL (IQC)

Not applicable

IX. SAFETY PRECAUTIONS

Always consider all used materials as infectious and discard appropriately.

X. REFERENCE

1. Laboratory diagnosis of Buruli ulcer: A WHO Manual for Health-care providers (edited by: Françoise Portaels) 2014. Available at *https://apps.who.int/iris/handle/10665/111738*; accessed on 28-11-19

XI. READING AND UNDERSTANDING LIST

NAME OF PERSONNEL	DATE	SIGNATURE

Annex 1: List of material and reagent for the extraction and purification of DNA from *M. ulcerans*

Name	reference	commentary
A manual register	Not applicable	Obtained locally
Sterile water	Not applicable	Not applicable
Genolyse kit	51610, Hain LifeScience	To be provided by BU LABNET
QIAquick purification kit	28106, QIAGEN	For biopsy samples. To be provided by BU LABNET
Vortex	Not applicable	Currently used in respective labs
Pipette	Not applicable	Currently used in respective labs
Filter tips	Will be provided by the BU LABNET, based on pipette information by labs	Labs will maintain currently used pipettes
Gloves	Not applicable	Non powdered
Disposable lab coat	Not applicable	Long sleeves/full length
Centrifuge	Any model that can hold 1.5ml tubes and spin up to 12000g	12000g 1.5ml
Dry water-bath	Not applicable	Currently used in respective labs
-20ºC Freezer	Not applicable	To store DNA eluate (long term)
Refrigerator 2-8ºC	Not applicable	To store DNA eluate (short term)
Waste container	Not applicable	Leak proof
