# Supplementary material for: A combined effort of 11 laboratories in the WHO African region to improve quality of Buruli ulcer PCR diagnosis: The “BU-LABNET”
Source: PLoS Negl Trop Dis. 2022 Nov 4;16(11):e0010908. doi: 10.1371/journal.pntd.0010908 (PMC9668193; doi:10.1371/journal.pntd.0010908)
Supplement: S5 Data — (PDF) [file pntd.0010908.s005.pdf]

Standard Operating Protocol 4:
QPCR preparation without Internal Positive Control

Author	Reviewer	Authorizer
Numfor Hycenth<br>Estelle Marion<br>Sara Eyangoh	BU Lab network members	Advisory Board

ABREVIATIONS

BU	Buruli Ulcer
PCR	Polymerase Chain Reaction
QPCR	Real-time Polymerase Chain Reaction
Ct	Cycle Threshold
WHO	World Health Organization

Table of Contents

I. PURPOSE 2

II. APPLICATION DOMAIN 2

III. ASSOCIATED DOCUMENTS 2

IV. TYPE OF SAMPLES 2

V. REAGENTS AND CONSUMABLES 2

VI. EQUIPMENT 2

VII. PROCEDURE 2

VIII. INTERNAL QUALITY CONTROL (IQC) 3

IX. SAFETY PRECAUTIONS 3

X. REFERENCE 3

XI. READING AND UNDERSTANDING LIST 4

Annex 1: List of material and reagent 5

Annex 2: Worksheet: QPCR mix calcul and plate plan 6

Standard Operating Protocol 4:
QPCR preparation without Internal Positive Control

I. PURPOSE

This Standard Operating Protocol (SOP) aims at presenting the different steps involved in preparing QPCR for *Mycobacterium ulcerans* without the internal positive control.

II. APPLICATION DOMAIN

To be applied to all laboratory members of the BU LAB Network for the PCR diagnosis of Buruli ulcer

III. ASSOCIATED DOCUMENTS

Worksheet: QPCR mix calcul and plate plan (annex 2)

IV. TYPE OF SAMPLES

► Swabs are used for sampling of opened lesions.

► Fine needle aspiration (FNA) is used for sampling of closed lesions.

► Biopsy is not recommended for case confirmation of Buruli ulcer.

V. REAGENTS AND CONSUMABLES

See list in annex1

VI. EQUIPMENT

See list in annex 1

VII. PROCEDURE

7.1 Prepare the experiment

1. Complete the plate plan of the worksheet for each new amplification with samples
2. Count the number of amplifications needed to be prepared. Important, count always 3 supplemental reactions to have enough mix at the end.
3. Calculate and fill in the table of PCR mix preparation (annex 2)
4. Switch on the thermocycler and fill in the plate plan

7.2 PCR mix preparation

Under a clean PCR hood

1. Wear gloves and a disposable blouse dedicated to this space.
2. Defrost the following reagents: QPCR master mix / primers and probe/an aliquot of sterile water
3. Probe dilution: 10-fold dilution before using
   - centrifuge the defrost tube
   - in a new screw cap tube, add 18µl of water
   - add 2µl of probe
   - vortex slowly and centrifuge for a few seconds

4. Primers dilution :
   - centrifuge the two aliquots of primer tubes
   - add directly 95µl of water into each tube
   - vortex slowly and centrifuge for a few seconds

7.3 PCR mix preparation :

1. follow the quantity of water, master mix, probe and primers calculated in the worksheet
2. prepare a new screw cap tube and mix the master mix by reversal
3. always start by pipetting water, then the 2 primers and the probe. Finish by adding the master mix.
4. mix the tube by reversal and centrifuge it few seconds
5. prepare a rack with 8 strip tubes
6. transfer 20µl of the mix in each tube
7. do not close the tubes

7.4 PCR mix and samples

1. On a dedicated bench, bring the rack with the PCR strip, the patient samples and plasmid
2. Add 5µl of patient samples following the plate plan
3. Close the patient strips
4. Prepare the standard range of plasmid DNA in screw cap tubes
   - Defrost an aliquot of 10µl tube at 1E8 bact/ml.
   - Add 90µl of water directly in the tube = first point of the standard curve= 1E7 bact/ml.
   - Add 45µl of water in 5 new screw cap tubes.
   - Perform cascade dilution by pipetting 5µl of the first tube and mix it to the 45µl tube.
   1E7 bact/ml, 1E6 bact/ml, 1E5 bact/ml, 1E4 bact/ml, 1E3 bact/ml, 1E2 bact/ml,
   - Add 5µl of plasmid DNA in strip tubes following the plate plan.
   - close the strips.

7.5 Amplification

1. Centrifuge the strips and place it into the thermocycler.
2. Run the program of amplification

7.6 PCR Analysis

1. check the two negative controls: extraction and mix preparation
2. check the standard curve: ensure that Ct values and R2 are OK
3. limit of detection for human: <35 cycles.
4. calculate the number of bacilli/ml for positive samples.
5. write the result in the manual registration book and in the worksheet for the clinician.
6. disseminate the results following the procedure validated in your country (email, whatsapp, etc)

VIII. INTERNAL QUALITY CONTROL (IQC)

No Internal Positive Control

IX. SAFETY PRECAUTIONS

Always consider all used materials as infectious and discard appropriately.

X. REFERENCE

1. Laboratory diagnosis of Buruli ulcer: A WHO Manual for Health-care providers (edited by: Françoise Portaels) 2014. Available at *https://apps.who.int/iris/handle/10665/111738*; accessed on 28-11-19

XI. READING AND UNDERSTANDING LIST

NAME OF PERSONNEL	DATE	SIGNATURE

Annex 1: List of material and reagent for QPCR

Name	reference	commentary
A worksheet for PCR mix preparation	Use worksheet disseminated by BU lab network	Worksheet version Jan 2010 validated
QPCR master mix	**HOT FIREPol Probe qPCR Mix Plus** (*Solis BioDyne, new mix candidate, by Dutscher)*	To be provided by BU LABNET
Primers and probe	New primers and probes will be used. To be provided by BU LABNET	To be provided by BU LABNET
Is2404 plasmid	(IS2404)<br>n° 30-8606-01 (GenExpress)	To be provided by BU LABNET
Sterile water	Not applicable	Not applicable
8 strip-tubes and caps	Not applicable	Currently used in respective labs
1.5ml microtube with screw-cap	For example: 39289 (Dutscher)	Ensure that cap is attached to the tube
Vortex mixer	Not applicable	Currently used in respective labs
Pipette	Not applicable	Currently used in respective labs
Filter tips	Will be provided by the BU LABNET, based on pipette information by labs	Labs will maintain currently used pipettes
Gloves	Not applicable	Non powdered
Disposable lab coat	Not applicable	Long sleeves/full length
QPCR machine	Not applicable	QPCR machine currently used in respective labs
Waste container	Not applicable	Leak proof

Annex 2: Worksheet: QPCR mix calcul and plate plan

UNDER CLEAN PCR HOOD

qPCR MIX PREPARATION

Note : qPCR assay used FAM-BHQ1 (5'-3') labeled probe

	H2O PPI	Probe/Primers	
Probe IS2404_TP (5 pmol/µL)	**18 µL**	**2 µL** (50 pmol/µL)	--> In a new screw cap tube
Primers IS2404_TF/TR (5 pmol/µL)	**95 µL**	**5 µL** (100 pmol/µL)	--> Add directly 95 µL of water in the tube

- Vortex slowly and centrifuge few second
- Mix the master mix by reversal

Important : Prepare the mix for = 14 + (S*2) + 2 + 3 **S = 12 patients samples**

= standard range (**14**) + patients samples in duplicate (**S x 2**) + extraction control (Ctrl) in duplicate (**2**) + **3**

Component	1 tube	43 tubes	Final [ ]
**5X Hot FirePol Probe qPCR mix**	5 µL	**215 µL**	1X
**H2O PPI**	11.5 µL	**494.5 µL**	
**IS2404_TF 5 pmol/µL**	1.5 µL	**64.5 µL**	300 nM
**IS2404_TR 5 pmol/µL**	1.5 µL	**64.5 µL**	300 nM
**IS2404_TP 5 pmol/µL**	0.5 µL	**21.5 µL**	100 nM
**Total Volume**	20 µL	**860 µL**	

- Mix the tube by reversal and centrifuge few seconds
- Annotate strip tubes
- Transfer 20 µL of the mix in each tube

ON A DEDICATED BENCH

PLATE SET UP

	1	2	3	4	5	6	7	8	9	10	11	12
**A**	1.E+07	1.E+07	S1	S1								
**B**	1.E+06	1.E+06	S2	S2								
**C**	1.E+05	1.E+05	S3	S3								
**D**	1.E+04	1.E+04	S4	S4								
**E**	1.E+03	1.E+03	S5	S5								
**F**	1.E+02	1.E+02	S6	S6								
**G**	Blank	Blank	S7	S7								
**H**	Ctrl	Ctrl	S8	S8								

- Bring the rack with the PCR strip, the patient samples and plasmid
- Add 5 µL of patient samples, following the plate set up
- Close the patients strips
- Prepare the standard range of plasmid DNA in screw cap tubes :
  . Defrost an aliquot of 5 µL at 1E8 bact/ml
  . Add directly 45 µL of water in the tube and centrifuge few seconds
  . Add 45 µL of water in 5 new screw cap tubes
  . Perform cascade dilution by pipetting 5 µL of the first tube and mix it to the 45 µL tube
- Add 5 µL of plasmid DNA or 5 µL of water (blank) in strip tubes, following the plate plan
- Close the strips

THERMAL PROFIL SET UP

Cycle step	T°C	Time	Cycles
**Initial activation**	95°C	10 min	1
**Denaturation**	95°C	15 s	40
**Annealing/Elongation**	60°C	1 min	
